# High-resolution, high-throughput imaging with a multibeam scanning electron microscope

**DOI:** 10.1111/jmi.12224

**Published:** 2015-01-27

**Authors:** AL EBERLE, S MIKULA, R SCHALEK, J LICHTMAN, ML KNOTHE TATE, D ZEIDLER

**Affiliations:** * Carl Zeiss Microscopy GmbHOberkochen, Germany; † Max-Planck-Institute for Medical ResearchHeidelberg, Germany; ‡ Department of Molecular and Cell Biology, Harvard UniversityCambridge, Massachusetts, U.S.A.; § Graduate School of Biomedical Engineering, University of New South WalesSydney, Australia

**Keywords:** High-throughput imaging, multibeam, parallel data acquisition, scanning electron microscopy

## Abstract

Electron–electron interactions and detector bandwidth limit the maximal imaging speed of single-beam scanning electron microscopes. We use multiple electron beams in a single column and detect secondary electrons in parallel to increase the imaging speed by close to two orders of magnitude and demonstrate imaging for a variety of samples ranging from biological brain tissue to semiconductor wafers.

**Lay Description:**

The composition of our world and our bodies on the very small scale has always fascinated people, making them search for ways to make this visible to the human eye. Where light microscopes reach their resolution limit at a certain magnification, electron microscopes can go beyond. But their capability of visualizing extremely small features comes at the cost of a very small field of view. Some of the questions researchers seek to answer today deal with the ultrafine structure of brains, bones or computer chips. Capturing these objects with electron microscopes takes a lot of time – maybe even exceeding the time span of a human being – or new tools that do the job much faster. A new type of scanning electron microscope scans with 61 electron beams in parallel, acquiring 61 adjacent images of the sample at the same time a conventional scanning electron microscope captures one of these images. In principle, the multibeam scanning electron microscope’s field of view is 61 times larger and therefore coverage of the sample surface can be accomplished in less time. This enables researchers to think about large-scale projects, for example in the rather new field of connectomics. A very good introduction to imaging a brain at nanometre resolution can be found within course material from Harvard University on http://www.mcb80x.org/# as featured media entitled ‘connectomics’.

## Introduction

Electron microscopy is commonly used to image biological samples, enabling acquisition of high-resolution images from small sample regions, typically in the range of several micrometres. However, there is an increasing need for large area imaging or even volume imaging of biological tissues at nanoscopic resolution comprising billions of pixels. An example is connectomics, an emerging field in neuroscience aimed at comprehensively reconstructing neural circuits (Lichtman & Denk, 2011; Helmstädter *et al*., 2013) and analysing extended cellular structures (Holcomb *et al*., 2013). To image surfaces, such as in the serial block-face technique (Denk & Horstmann, 2004) or with ultrathin sections on a solid substrate (Micheva & Smith, 2007; Horstmann *et al*., 2012), scanning electron microscopes (SEMs) need to be used, which conventionally acquire an image one pixel at a time. This limits the data acquisition rate and, in turn, the sample size that can be imaged within a reasonable amount of time. As an example, mapping a 1 mm cube of tissue with an isotropic voxel size of 4 nm will result in almost 16 petabytes of data. Data acquisition at 20 MHz would require a total acquisition time of almost 25 years, even before taking into account overhead times such as those due to stage movements.

Another application is quality control, such as is needed for wafer production, where we need to detect nanometre-sized particles and defects (Patterson *et al*., 2012). A similar protocol is followed in medical research where histological samples are searched for characteristic motifs, antibodies or nanoparticles (Kaiser *et al*., 2013). Due to throughput limitations, these methods are confined to analyses of a small fraction of the sample surface, thereby increasing the number of ‘false negative’ events.

Finally, organs and tissues exhibit architectures which emerge hierarchically from cell-scale events culminating at the macroscale and resulting in ‘smart’ or stimuli-responsive properties (Evans *et al*., 2013). Application of experiments and computer models in parallel enables mechanistic elucidation of these system properties (Knothe Tate, 2011; Moore *et al*., 2014). To date, different imaging methods had to be used to bridge the gap between different length scales. The capability to image the structure of organs and tissues seamlessly across length scales using high-throughput electron microscopy is expected to facilitate understanding of functional implications across length scales.

The above-mentioned applications show that there is a strong need for high-throughput electron microscopy that can image large areas or volumes at high resolution. However, because scanning electron microscopy is widely thought of as an inherently low-throughput technique, the SEM may not even be considered for applications that require high-throughput imaging. Here, we demonstrate a multibeam SEM with a throughput increase by almost two orders of magnitude.

## Throughput limitations of single-beam SEMs

The signal-to-noise ratio in an SEM image depends on beam current, pixel dwell time, sample contrast and detection efficiency. It has to be larger than a minimum signal-to-noise ratio to make features in the data set – the signal – visible sufficiently well against background noise (Bright *et al*. 1998). If we want to increase throughput by decreasing the pixel dwell time, we have to increase the beam current to maintain the signal-to-noise ratio if all other parameters remain unchanged. Increasing the beam current will lead to increasing Coulomb interactions between the electrons, thereby blurring the electron beam and reducing the resolution. Moreover, we cannot operate electron detectors faithfully at arbitrarily high rates because electrons of different energies reach the detector at different times, thereby limiting the effective detector bandwidth. Another limitation of the bandwidth of efficient secondary electron (SE) detectors is determined by signal decay time, such as occurs at a scintillator.

## Circumventing the single-beam limitations with a multibeam approach

**Figure 1:**
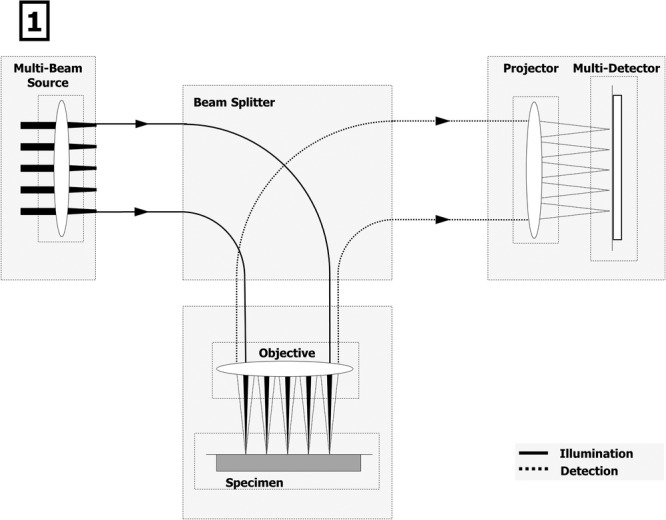
The multibeam SEM uses multiple beams in parallel to image a hexagonal sample area 100-μm wide. Primary electrons (solid lines, left) are focused onto the specimen and separated by a beam splitter from the secondary electrons (dotted lines, right) which are detected simultaneously. All electron beams form many individual images which are then merged into a single, large area micrograph.

The multibeam SEM currently uses 61 electron beams in a single column and one dedicated detector for each beam to alleviate Coulomb interaction limitations and bypass the detector bandwidth limit. The principle of operation is depicted in Figure1: A multiple beam electron source produces a regular array of electron beams that are imaged onto the sample, forming a pattern of 61 primary electron foci. The array of primary beams is arranged in a hexagonal pattern to minimize electron optical aberrations. The SEs that emanate from each primary electron spot are imaged onto a multidetector with one detection unit for each electron beam. A magnetic sector field separates primary electron and SE beams. The electron beams are scanned over the sample, and the SE signal is recorded for each scan position as in conventional SEMs. One single scanning pass thus produces multiple images in parallel yielding a complete image of the sample region underneath the primary beam array. A number of options for producing an array of electron beams has been demonstrated (Platzgummer *et al*., 2013 and references therein). More options to perform imaging with a multibeam electron microscope have also been demonstrated (Mohammadi-Gheidari *et al*., 2010; Mohammadi-Gheidari & Kruit, 2011; Enyama *et al*., 2014).

The total possible detector bandwidth of the multibeam SEM is the single detector bandwidth times the number of beams, in this case 1.22 GPixel/s. The Coulomb interaction is lower than in a single-beam configuration, as the charge is distributed across many beams and therefore spread over a larger volume inside the electron optical column. The data acquisition computer system is highly parallelized to accommodate the large data acquisition rates and enable comparably low-bandwidth storage solutions.

## Application examples of the multibeam SEM

We demonstrate that the speed increase is achieved with a number of samples from the above-mentioned applications and that the multibeam SEM is compatible with the corresponding sample preparation methods. Typical landing energies of the multibeam electron microscope are 1–3 keV, typical pixel sizes are 4–10 nm. The electron optical setup has been chosen such that the distance between single beams is 12μm. The number of pixels per image in *x* and *y* and the pixel size are independent of the beam distance and have been chosen such that a small overlap between the images is obtained, which requires an aspect ratio of about 0.866 between *x* and *y*. In principle, other pixel sizes and aspect ratios can be chosen, but might result in too large overlaps or no overlap at all. In the following examples, the 61 images have been merged into one large image that contains up to 500 MPixel over a hexagonal area approximately 100 μm wide.

**Figure 2:**
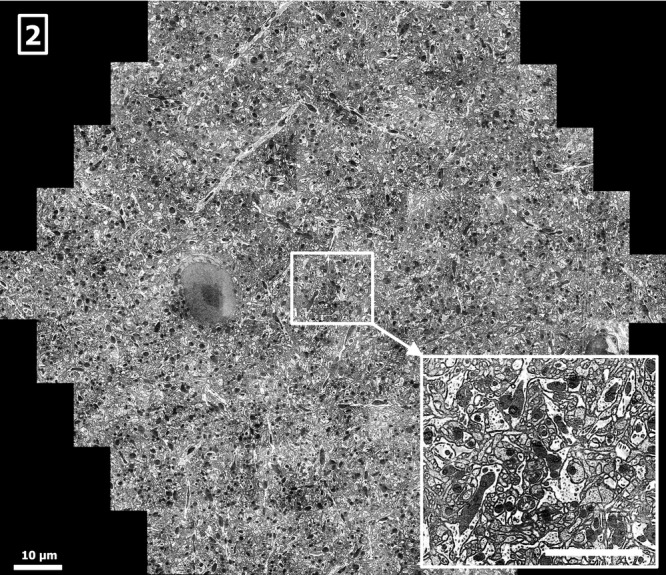
Cerebral cortex of mouse brain (block-face), sample by Winfried Denk and Shawn Mikula, Max Planck Society, showing unmyelinated neuronal and glial processes and a neuronal nucleus (left of centre), acquired by the multibeam SEM at 0.45 GPixel/s and 3.8 nm pixel size, 26 nA total current, 270 electrons per pixel, scale bar: 10 μm. A 1–2 nm coat of palladium has been evaporated onto the block-face to dissipate charging (Titze & Denk, 2013). Within the cellular processes, mitochondria, microtubules, synapses and endoplasmic reticulum are visible. Inset lower right: 12 μm × 10 μm single-beam subimage, detail of the full multibeam image, scale bar: 5 μm.

**Figure 3:**
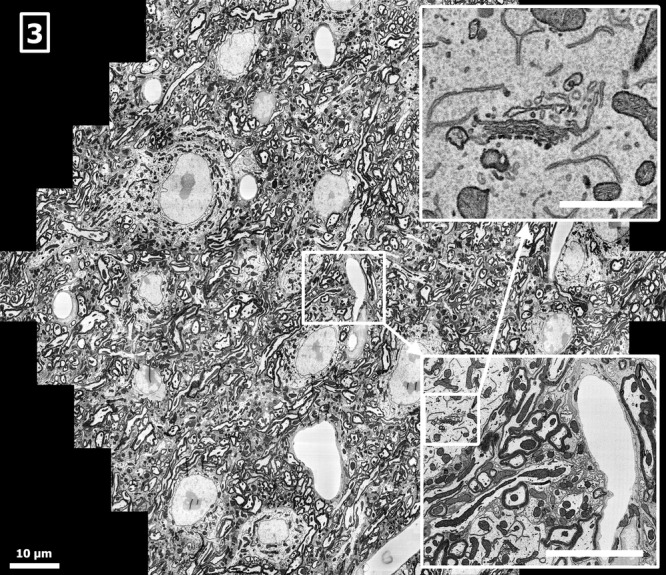
Cortex of mouse brain (serial ultrathin section), sample by Jeff Lichtman and Richard Schalek, Harvard University, showing myelinated axons, plasma membranes, cell somata and dendrites, acquired by the multibeam SEM at 0.45 GPixel/s and 3.8 nm pixel size, 26 nA total current, 270 electrons per pixel, scale bar: 10 μm. Sample charging has been mitigated by placing the thin sections on a conductive surface such that no additional conductive coating is required. Within the cells, dendrites and axons, organelles such as mitochondria and endoplasmic reticulum are visible. Inset lower right: 12 μm × 10 μm single-beam subimage, detail of the full multibeam image, scale bar: 5 μm. Inset upper right: 3 μm × 2.6 μm detail of the single-beam subimage, scale bar: 1μm.

For the large-volume investigation of biological tissues, an example is the volumetric reconstruction of a macroscopic volume of mouse brain tissue (Lichtman & Denk, 2011). To obtain such data, a number of solutions for sectioning and imaging exist (Briggman & Bock, 2012). For example, a slice of several nanometres in thickness can be removed from the sample surface using an in-chamber microtome. The freshly exposed sample surface is then imaged with an SEM. Repeating this process many times yields a data set of the entire volume (Denk & Horstmann, 2004). It is also possible to collect ultrathin sections on a flat substrate and subsequently image them with an SEM (Micheva & Smith, 2007; Horstmann *et al*., 2012). The multibeam SEM is compatible with both methods, as demonstrated in Figures2 and 3. Figure2 shows a subregion from a coronal block-face of an osmium-stained mouse brain (Mikula *et al*., 2012; Mikula & Denk, 2014, in preparation) taken at approximately Bregma 1.5 mm. Figure3 shows a multibeam SEM acquisition from a serial section taken from an osmium-stained mouse brain block. The sample preparation with an automatic section collection device has been described in Hayworth *et al*. (2006) and Tapia *et al*. (2012). The whole experimental setup is designed such that a high degree of automation enables the reliable acquisition of large quantities of EM data (Hayworth *et al*., 2014). We expect the signal in Figures2 and 3 to be composed of SE1 and SE2 electrons mainly (Reimer, 1993; Cazaux, 2004) with a penetration depth of the SE1 contribution of about 10–20 nm (Seiler, 1983) and a slightly higher penetration depth for the SE2 contributions (Hennig & Denk, 2007). The SE1 contributions are known to be dependent on surface topology (Griffin, 2011), such as knife marks and section folds. These defects are addressed by optimizing the sample preparation such that the frequency of occurrence is minimized and by data postprocessing.

**Figure 4:**
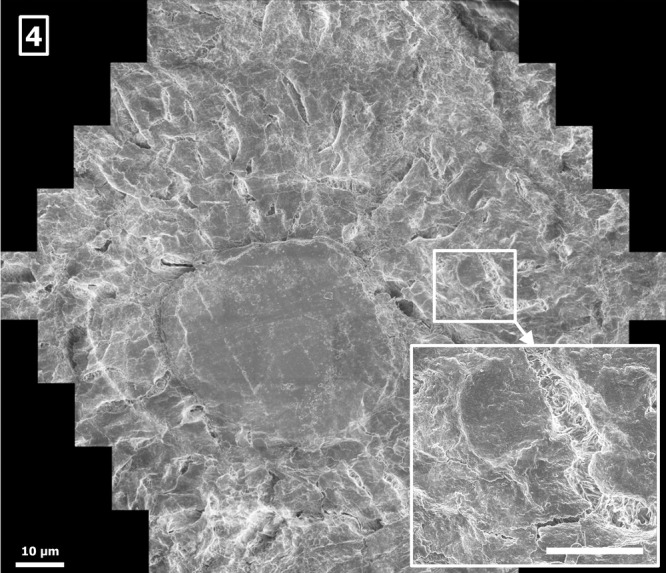
Femoral neck (PMMA-embedded and polished block-face), sample by Melissa Knothe Tate, University of New South Wales, and Ulf Knothe, Cleveland Clinic, showing an osteon comprising a bone capillary surrounded concentrically by osteocytes, acquired by the multibeam SEM at 0.18 GPixel/s and 11.3 nm pixel size, 40 nA total current, 420 electrons per pixel, scale bar: 10 μm. Inset lower right: 12 μm × 10 μm single-beam subimage, detail of the full multibeam image, showing one osteocyte, scale bar: 5 μm.

Imaging tissues or organs over several length scales results in samples of up to several centimetres diameter. Sample preparation requires special attention to provide a sufficiently uniform sample fixation and surface smoothness. Figure4 shows the multibeam SEM image of a section of a human femoral neck collected per IRB (institutional review board) protocol guidelines, and normally discarded in the course of hip replacement surgery (Chang *et al*., 2014). Tissues are sectioned and prepared for undecalcified histology with bulk embedding in polymethylmethacrylate. After curing of the resin, the surface is trimmed and smoothed for SEM inspection of the block-face (Knothe Tate, 2015, in preparation).

**Figure 5:**
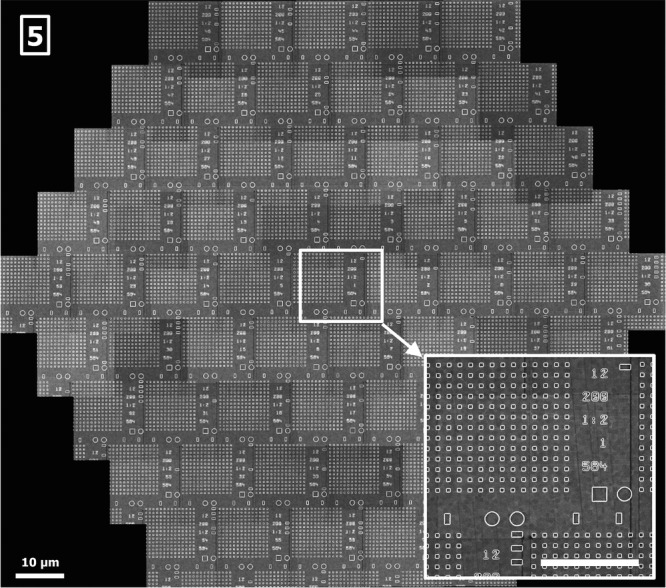
Test chip showing a hexagonal arrangement of calibration structures for tool adjustments. The structures are printed in an e-beam direct write lithography process with a high placement precision, etched in SiO_2_ on a Si-substrate, and finally coated with a completely conductive layer, scale bar: 10 μm. Pixel size was 3.8 nm, acquisition speed 0.72 GPixel/s, 40 nA total current, 210 electrons per pixel. Inset lower right: 12 μm × 10 μm single-beam subimage, detail of the full multibeam image, scale bar: 5 μm.

All composite images (Figs.5) show sufficient contrast in all subimages and the resolution of all subimages varies by only a few percent. The crosstalk between adjacent beams is below 1%. To make the imaging capabilities of the multibeam SEM accessible to large-scale applications, it is desirable to automate the data acquisition process.

## Operating a multibeam SEM

Because the fundamental principles of an SEM are conserved in the multibeam SEM, operation is in many respects identical to operating a single-beam SEM. The main difference is that a multibeam SEM requires a higher degree of automation during adjustment, such as measuring and controlling parameters (e.g. focus, stigmation, relative beam position) for all beams, which is difficult and time consuming for a human operator. As an example, the illumination optics of a multibeam SEM typically consist of a number of electron optical lenses. During general alignment, if focus adjustment is needed, changing the excitation of one of the lenses results not only in the change of focus, but also in a change of beam pitch at the sample. If the excitation of a magnetic lens is changed, not only will the beam pitch be altered, but also the rotation of the beam positions around the central beam. Thus, adjusting one parameter such as focus requires that multiple lens settings must be adjusted accordingly. This is most conveniently solved by an algorithm in connection with an appropriate test sample. One possible test sample is shown in Figure5. It contains test structures written onto a wafer, which are suitable for automated alignment and calibration of a multibeam SEM. During data acquisition, for very small changes of focus (and stigmation), using just one lens (or stigmator) for automatic alignment is usually sufficient, and may be performed fast on any type of sample with sufficient structural information. The frequency of the automatic alignment depends on sample properties such as sample flatness, as the drifts of the multibeam SEM are small.

Furthermore, as large data sets are automatically acquired and evaluated by algorithms, many images are likely to never be inspected by a human being. Therefore, a large degree of automation is required during the data acquisition process as well. We will demonstrate this at the example of wafer stage movements for seamless acquisition of sample surfaces.

Acquiring large areas with a multibeam SEM is performed in the same manner as with a single-beam SEM: The sample is mounted onto a stage such that multiple fields of view (FoV) cover the areas to be imaged. With a single-beam SEM, an efficient tessellation of the region of interest is possible for any rotation of the SEM scan direction, that is, orientation of the FoV of the single beam, as the movement of the sample can be chosen such that the area is completely covered with only small overlaps of the FoV being required. With a multibeam SEM, the tessellation has to take into account the relative beam positions of the multiple beams at the surface of the sample. This produces two constraints: (i) In order to produce an efficient tiling of the sample surface, the direction of the scan rotation of the scanning system that defines the axes of all single images has to coincide with the direction of one beam to one of its (four or six) nearest neighbours. In doing so, the FoV for each beam can be chosen equal to the beam pitch and thus be minimized. If the scan differs from this beam orientation, a larger FoV at the same beam pitch must be chosen to completely image the area of interest, which means that a certain fraction of the surface is scanned more than once, leading to a reduction of throughput. Setting the optimal relation between the two directions is possible by either choosing a scan rotation according to the beam positions at the sample, or, if a special scan rotation is desired, by rotating the beam positions accordingly. The overlap between adjacent single-beam images ensures proper stitching of the images to below a few pixels within one hexagonal field of view. (ii) The tessellation of the region to be scanned has to take into account the arrangement of beams at the sample surface. For example, a hexagonal beam arrangement produces a hexagonal-like shaped total FoV for the entirety of all beams. A complete tiling of the surface with minimal overlap is then possible using adapted stage positions. As before, fulfilling these constraints is most conveniently solved by an algorithm.

## Conclusions

We have shown that the multibeam SEM is capable of high-throughput imaging (approaching 1 GHz) of a variety of samples of different compositions and contrast mechanisms, leading to a remarkable reduction in image acquisition time compared to a conventional single-beam SEM. Thus, we expect that the multibeam SEM will open up new fields of research where nanometre-resolution imaging over macroscopic areas and volumes in the range from millimetre to centimetre (Marx, 2013) are of utmost importance.
